# CMV, B and C hepatitis among multi-transfused hereditary hemolytic Anemia children: an updated Egyptian experience

**DOI:** 10.1186/s13052-021-01072-x

**Published:** 2021-05-26

**Authors:** Laila M. Sherief, Seham M. Ragab, Mohamed A. Helwa, Naglaa M. Kamal, Mona R. Afify, Rasha T. S. Mohammed, Ghada Abd Elmoniem Mokhtar, Hanan S. Sherbiny

**Affiliations:** 1 Pediatrics & Pediatric Hematology oncology, Faculty of Medicine, Zagazig University, Zagazig, Egypt; 2 Pediatrics & Pediatric Hematology oncology, Faculty of Medicine, Menoufia University, Menoufia, Egypt; 3 Clinical Pathology, Faculty of Medicine, Menoufia University, Menoufia, Egypt; 4 Pediatrics and Pediatric Hepatology, Faculty of Medicine, Cairo University, Cairo, Egypt; 5 Medical Microbiology & Parasitology, Faculty of Medicine, University of Jeddah, Jeddah, 21589 Saudi Arabia; 6 Medical Microbiology & Immunology Department, Faculty of Medicine, Zagazig University, Zagazig, Egypt; 7 Pediatrics, Faculty of Medicine, Zagazig University, Zagazig, Egypt; 8 Pediatric Department, Collage of Medicine, University of Bisha (UB), Bisha, Kingdom of Saudi Arabia

**Keywords:** Seroprevalence, Hepatitis C virus, Hepatitis B virus, Cytomegalovirus, Hereditary hemolytic Anemias, Transfusion hepatitis

## Abstract

**Background and objectives:**

Regular blood transfusion has improved the overall survival and quality of life for patients with hereditary hemolytic anemias. Nevertheless, it carries a real risk of acquisition of blood-borne virus infections, especially viral hepatitis. The purpose of the current study is to present an Egyptian update on blood-borne hepatitis C & B viruses (HCV & HBV) and cytomegalovirus (CMV) among multi-transfused Egyptian children with hereditary hemolytic anemias, especially after implementation of national preventive programs in Egypt.

**Patients and methods:**

All pediatric patients with hereditary hemolytic anemias who have regular follow-up and received frequent blood transfusion at the Pediatric Hematology Units, Menuofia and Zagazig Universities Hospitals, Egypt, during the study period, were recruited. They were tested for hepatitis B surface antigen (HBVsAg), hepatitis C antibody (HCVab), and CMV immunoglobulin M (IgM) serology. Those with positive results were confirmed by real-time polymerase chain reaction (PCR).

**Results:**

Four hundred and seventy-seven hereditary hemolytic anemia patients fulfilled the study inclusion criteria. Their ages ranged from 2 to 18 years, 54.9% of them were males. Seroprevalence of HCVab and CMV-IgM were (14.7% & 6.7% respectively) and they were confirmed by PCR. None of the studied cases were HBVsAg positive. Seropositivity for HCV was significantly associated with older age of the patients, higher transfusion frequency, longer disease duration, and higher mean serum ferritin.

**Conclusion:**

HCV followed by CMV infections still represent a significant problem for patients with hereditary hemolytic anemias. Nationwide plans should be taken to ensure meticulous and highly sensitive methods of blood screening before transfusion. On the other hand, it seems that HBV compulsory vaccination had succeeded to eliminate HBV infection.

## Core tip

Transfusion hepatitis continue to be a major problem in multi-transfused children. In this study, we studied the magnitude of this problem in pediatric hemolytic anemia patients. HCV and CMV were seropositive in 14.7 and 6.7% of our patients respectively, confirmed by PCR. None of them were HBVsAg positive. Older age of the patients, higher transfusion frequency, longer disease duration and higher mean serum ferritin were the main associated factors.

## Introduction

Hereditary hemolytic anemias include diverse groups of phenotypically and genetically heterogeneous disorders that result in an increased rate of RBC destruction [1]. The most common of these disorders are hemoglobinopathies, hereditary spherocytosis and glucose-6-phosphate dehydrogenase, which affect millions of people worldwide [2].

Although regular blood transfusion has improved the overall survival and quality of life in patients with hereditary hemolytic anemias, it carried a real risk of acquisition of blood-borne virus infections, especially viral hepatitis [3]. Hepatitis C virus (HCV) infection is a major blood-borne infection, with silent epidemiology, that has reached pandemic proportion**.** Globally, it is reported that 210 million patients are chronically infected, whereas, 3–4 million individuals are newly infected yearly [4]. In 2012, Egypt had 14.7% seroprevalence of HCV in adults [5] and 5.8% in children [6]. HCV prevalence among multi-transfused Egyptian patients ranged between 10 and 55% [7]. Hepatitis B virus (HBV) infection is a significant global problem with more than 2 billion people infected, including an estimated 400 million chronically infected cases [8]. The prevalence of HBV surface Antigen (HBVsAg) in Egypt was determined as 2–8% of all population, with nearly 2 to 3 million Egyptians are chronic carriers. As high as 29% HBVsAg positive patients were reported among multi-transfused children in Egypt [9]. Transfusion-transmitted cytomegalovirus (CMV) infections is usually asymptomatic [10]. On the other hand, it can cause serious, and potentially morbid complications in immunocompromised blood recipients, including splenectomized individuals [11]. Surgical or autosplenectomy largely presents a state of immune deficiency. Therefore, those patients could be considered at risk for serious CMV infections [11]. Unfortunately, in Egypt, there is lack of central surveillance system for assessment of infectious diseases, like blood/blood products transfusion-transmitted infections.

Therefore, regional and periodic studies are the only ways to monitor trends of such illnesses. Egypt has implemented nationwide preventive & treatment strategies to combat viral hepatitis but currently no recent data on the impact of these measures on seroprevalence of transfusion-transmitted hepatitis viruses in Egypt. The aim of the current study is to update the status of blood born hepatitis viruses; HCV, HBV and CMV; in multi-transfused Egyptian children with hereditary hemolytic anemias especially after implementation of national preventive & treatment viral hepatitis strategies.

## Patients and method

All patients with transfusion dependent hereditary hemolytic anemia fullfilling the study inclusion and exclusion criteria from those attending the pediatric hematology Units of Menoufia University Hospital and Zagazig University Children’s Hospital, Egypt, were consecutively recruited during the period from November 2014 to November 2016.

### Inclusion criteria

1. Transfusion dependent hereditary hemolytic anemia (multi-transfused)
2. Age; < 18 years
3. On regular follow up with complete medical records
4. Negative for HCV, HBV and CMV pretransfusion

### Exclusion criteria

1. Anemia due to any cause other than hereditary hemolytic anemia
2. Any associated chronic disease which might necessitate frequent blood transfusion
3. Recently diagnosed hereditary hemolytic anemia patients who didn’t receive frequent blood transfusion

### Ethical approval

The study was approved by the research and ethical committees of Faculty of Medicine Menoufia and Zagazig Universities, Egypt.

### Informed consent

Written informed consents were obtained from the parents/legal guardian of the enrolled children for contribution of their children in the study and for publication.

### All enrolled patients were subjected to the following

I: Full history taking with especial emphasis on; age at diagnosis, presenting symptoms, detailed transfusion history [age of first blood transfusion, frequency and amount of blood with calculation of red blood cells (RBCs) transfusion index], and the intake of iron chelating agents (their types, duration and compliance).

II: Thorough general and systemic clinical examination.

Note that; body built was classified as under, normal or over body built by two methods. For infants up to the age of 2 years, Body mass index (BMI) was not assessed. Instead, the infants’ weight percentiles were compared to length percentiles and plotted on WHO charts. BMI charts were used for children between the ages of 2–18 years. BMI percentiles classified patients into; < 5th percentile (under), 5th–85th percentile (normal), 85th–95th percentile (over), and > 95th percentile (obese).

III: Laboratory investigations:

Five ml of venous blood were aseptically withdrawn from each patient before blood transfusion. The sample was divided into two tubes; 4 ml were transferred into plain tubes, let to stand to clot and centrifuged for 15 min at 3000 rpm for serological assay while one ml was transferred in EDTA tube for complete blood picture (CBC).

- Routine laboratory investigations including; CBC (Sysmex XN -1000 SA-01, Germany), liver function tests (Beckman coulter (synchron CX 9 ALX), clinical auto analyzer, USA) and serum ferritin; Elecsys Ferritin Electrochemiluminescence immunoassay for the in vitro quantitative determination of ferritin in human serum or plasma by using Cobase-411 device, Roche Diagnostics, Switzerland.
- HBVsAg; by using the Elecsys (Roche Diagnostics, Melan, Italy) which is a two-step sandwich chemiluminescent microparticle immunoassay (qualitative third generation enzyme immunoassay).
- HCV antibodies (HCVab); by Murex anti-HCV (version 4.0), DiaSorin S.p.A, Italy, which is antibody sandwich 3rd generation enzyme-linked immunosorbent assay (ELISA). Viral load was classified into three categories based on its level; low (< 60, 0000 IU/ml), intermediate (60,0000–80,00000 IU/ml) and high (> 80,00000 IU/ml) viral load [12].
- CMV Immunoglobulin M (CMV-IgM): by ELISA (Euroimmum kit, Germany)
  For seropositive HCV patients, quantitative real-time polymerase chain reaction (PCR) by COBAS Amplicor 2.0, Roche Molecular Diagnostics, Pleasanton, CA, USA (lower limit of detection of 10 IU/mL) to determine HCV-RNA load and genotype.
- In case of HBVsAg positive, serum HBV-DNA was tested using a standardized automated quantitative real-time PCR assay (COBAS TaqMan HBV test, Roche Diagnostics, Branchburg, NJ; detection limit 12 IU/ml).
- In those with CMV-IgM positive, applied biosystem real time PCR (StepOne TM Real-Time PCR System, Applied Biosystem Inc, USA) was applied for quantification of the virus after DNA extraction by using QlAamp DNA Blood Kit (Qiagen, Inc., Valencia, Calif.).

## Statistical analysis

Data was revised, coded and fed to statistical software IBM SPSS version 20. Normally distributed numerical data were presented as mean and standard deviation (mean ± SD), means of different variables were compared using independent sample t-test (unpaired) or paired t test according to the studied groups. Categorical data presented as frequency and percentage. Frequencies were compared by Chi-square (χ2) (independent proportions) test or McNemar test (dependent proportions). Skewed data were presented as median and range, Man Whitney test was used for analysis of numerical data, while Chi square test was used for analysis of categorical data. All statistical analysis was done using two tailed tests and alpha error of 0.05. *P* value less than or equal to 0.05 was considered statistically significant.

## Results

**Fig. 1:**
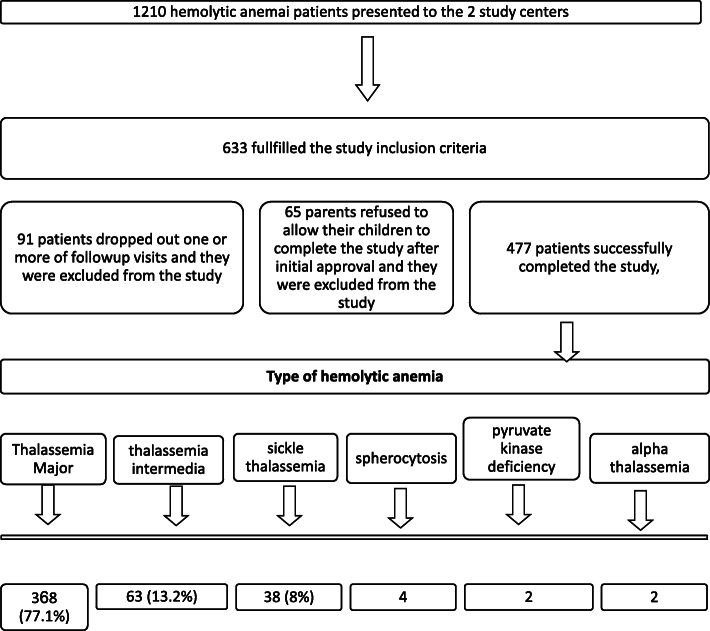
Flow Chart of Patients’ Enrollment

One thousand and two hundred ten (1210) patients with hereditary hemolytic anemias presented to the two study centers during the study period. Among them, 633 patients fulfilled the study inclusion criteria. Ninety-one patients dropped out some of the follow up confirmatory PCR tests, while 65 parents refused to allow their children to complete the study after initial approval. Both of those groups of patients were excluded from the study. Four hundred-seventy-seven (477) patients successfully completed the study, 368 (77.1%) of them were diagnosed as thalassemia major, 63 (13.2%) were thalassemia intermedia, 38 (8%) were sickle thalassemia, 4 patients had spherocytosis, 2 had pyruvate kinase deficiency and 2 were alpha thalassemia as displayed in the study flow chart (Fig. 1).

Reviewing of the initial medical records of all enrolled cases showed that; their mean age ± (SD) at diagnosis was 1.5 ± (1.5) ranged from 6 months to 9 years & 55% (262) were males. The main initial presenting clinical features were; pallor (100%), jaundice (92%), hepatomegaly (56%) and splenomegaly (78%). Pretransfusion initial screening for HBV, HCV and CMV was negative as a prerequisite for enrollment. Demographic characteristics and clinical presentations at initial diagnosis and at enrollment to the study were summarized in Table 1. Mean age at enrollment was 9.9 (±5.1), pallor and jaundice were the most frequent presentations, the majority of our cohort were underweight (59%), 33% of them had splenectomy, and the characteristic skeletal changes became more obvious as they get older (82%) especially among thalassemia major patients who were under-transfused.

**Table 1:** Demographic and Clinical Characteristics of the Study Population

Bio-Demographic Data	At Diagnosis (***N*** = 477)	At Enrollment (***N =*** 477)	McNemar Test (95% C.I)	***P*** value
**Study center**				
- Menofia No (%)	196 (41)			
- Zagazig No (%)	281 (59)			
**Gender**				
- Male	262 (55)			
- Female	215 (45)			
**Age (years)**				
- Mean (SD)	1.5 (±1.5)	9.9 (±5.1)	–	–
- Range	0.5–9	2–18		
**Body Built** No (%)				
- Underweight	137 (29)	280 (59)	29.98 (25: 34)	< 0.0001
- Normal	291 (61)	182 (38)	−22 (−26: −19)	< 0.0001
- Overweight	49 (10)	15 (3)	−7 (−9: −4.8)	< 0.0001
**Clinical Presentation** No (%)				
- Pallor	477 (100)	477 (100)		
- Jaundice	438 (91)	343 (72)	−9.85 (−12.5: −7)	< 0.0001
- Hepatomegaly	267 (56)	368 (77)	21 (17.5: 24.8)	< 0.0001
- Splenomegaly	372 (78)	259 (54) ^a^	–	
- Skeletal Changes	0 (0)	393 (82)	82 (79: 86.5)	< 0.0001

^a^165 patients (33%) are splenectomized

*SD* standard deviation, *CI* Confidence Interval

All our patients received multiple transfusions, with widely variable frequency (4–20 times) per year. Some patients started transfusion therapy as early as 6 months of age, while others were delayed up to 9 years, according to disease type and severity.

Chelation therapy is a pivotal protective therapy for all multi-transfused cases. In the studied cohort, 62.5% received oral chelation (42.8% deferasirox and 19.5% deferiprone) while only 12.2% received subcutaneous desferoxamine. Around 15.5% had very high serum ferritin and were treated with combination of desferoxamine and deferasirox. Unfortunately, 65% of the enrolled patients were “non-compliant” to chelation therapy. Transfusion and chelation therapy data are shown in Table 2.

**Table 2:** Blood Transfusion and Chelation Therapy Data of the Study Population

Transfusion and Chelation Variables	Data
**Disease duration (years)**	
- Mean (SD)	**8.1 (**±**4.9)**
- Range	**1–17**
**Age at 1st blood transfusion (years)**	
- Mean (SD)	**1.5 (**±**1.5)**
- Range	**0.5–9**
**Blood transfusion duration (years)**	
- Mean (SD)	**8.04 (**±**4.94)**
- Range	**1–17**
**RBC Transfusion Index (ml/kg/year)**	
- Mean (SD)	**183.14 (**±**48.5)**
- Range	**30–360**
**Transfusion frequency (times/year)**	
- Mean (SD)	**12 (**±**6)**
- Range	**4–20**
**Type of chelation No (%)**	
- Deferasirox	**204 (42.8)**
- Deferiprone	**93 (19.5)**
- Desferoxamin	**58 (12.2)**
- Combination) Deferasirox/Desferoxamine(	**72 (15.1)**
- None	**50 (10.5)**
**Compliance No (%)**	
- Compliant	**149 (35)**
-Noncompliant	**278 (65)**
**Chelation duration (years)**	
- Mean (SD)	**7.2 (**±**4.6)**
- Range	**0.5–17**

*RBC’s* Red Blood Cells, *No* Number, *SD* standard deviation

Results of general laboratory investigations, which were done at initial diagnosis and at enrollment to study, were tabulated and compared in Table 3. Significantly higher mean liver enzymes and serum ferritin were recorded at recruitment to the study (1–17 years disease duration) as compared to baseline values at diagnosis. Platelets count was also significantly higher at recruitment as compared to initial values especially among splenectomized patients.

**Table 3:** General Laboratory Data of the Study Population

Laboratory Investigation	At Diagnoses (***N =*** 477)	At Enrollment (***N =*** 477)	Paired–t-Test	***P*** value
**Hb (gm/dL)**				
- Mean (SD)	5.47 (±1.9)	7.63 (±0.9)	15.18	< 0.0001
- Range	5–9	5–10		
**WBC’s**				
- Mean (SD)	12.5 (±3.4)	13.2 (±7.9)	1.31	0.188
- Range	10–18	2.5–24.9		
**Platelets (10**^**3**^**/dL)**				
- Mean (SD)	257 (±85)	353.6 (±218)	6.66	< 0.0001
- Range	157–400	120–950		
**Serum Ferritin (ng/mL)**				
- Mean (SD)	85 (±16)	2935 (±2061)	23.02	< 0.0001
- Range	120–180	488–9661		
**AST (IU/L)**				
- Mean (SD)^a^	20 (±4)	48.2 (±15)	29.8	< 0.0001
- Range	12–56	19–442		
**ALT (IU/L)**				
- Mean (SD)^a^	12.5 (±8)	48.4 (±15)	33.7	< 0.0001
- Range	18–38	14–292		
**Total Bilirubin (mg/dL)**				
- Mean (SD)	1.1 (±0.3)	2.8 (±1.2)	22.6	< 0.0001
- Range	1.3–2.1	1.6–4.7		

^a^Trimmed Mean was used due to few outliners

*SD* standard deviation, *Hb* hemoglobin, *WBCs* white blood cells, *AST* aspartate transaminase, *ALT* alanine transaminase

Of paramount importance is the significantly higher HCV (14.7%) and CMV (6.7%) seropositivity among the study cohort at enrollment as compared to baseline negative results pretransfusion. Seventy patients were tested positive for HCVab and 32 patients were positive for CMV-IgM during the study. It is worth noting that twenty-two of the thirty-two CMV seropositive patients were positive for HCV as well.

None of our patients had seropositivity for HBV pretransfusion, at enrollment or during the study period.

All HCVab positive patients were confirmed by PCR apart from one patient who was under detection levels. According to HCV viral load, 17 patients had low viral load, 23 had moderate viral load and 29 had high viral load as shown in Table 4. Genotype 4 was the type detected in all our cohort. Similarly, CMV-IgM positive cases had been confirmed by CMV-PCR.

**Table 4:** HCV, HBV and CMV Status among the Study Cohort

Laboratory Investigation	At Diagnoses (***N*** = 477)	At Enrollment (***N*** = 477)	McNemar Test (95% C.I)	***P*** value
**HCVab No (%)**				
- Positive	0	70 (14.7)	14.2% 11.1–17.3%	< 0.0001
**HCV-PCR No (%)**				
- Under detection level	–	1 (1.4)		
- Low Viral load	–	17 (24)		
- Moderate Viral load	–	23 (33.6)		
- High Viral load	–	29 (41)		
**HBVsAg No (%)**				
- Positive	0	0	–	–
**CMV-IgM No (%)**				
- Positive	0	32 (6.7)	6.7% 4.46–8.95%	< 0.0001
**CMV-PCR No (%)**				
- Positive	0	32 (6.7)	–	–

*No* Number, *HCVab* Hepatitis C Virus antibody, *HCV-PCR* Hepatitis C Virus polymerase chain reaction, *HBVsAg* Hepatitis B surface antigen, *CMV* Cytomegalovirus

**Fig. 2:**
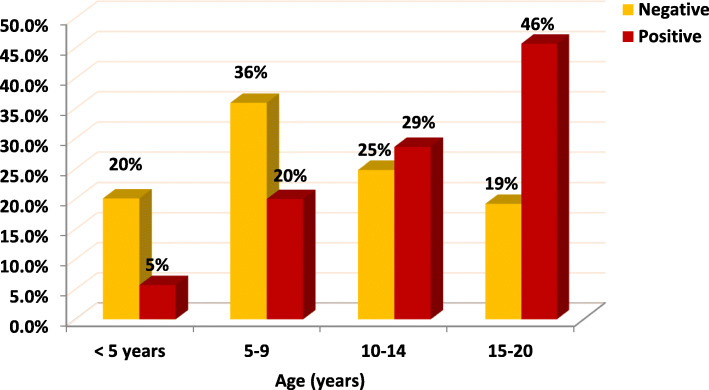
HCV Seropositive Rates among Different Age Groups

**Fig. 3:**
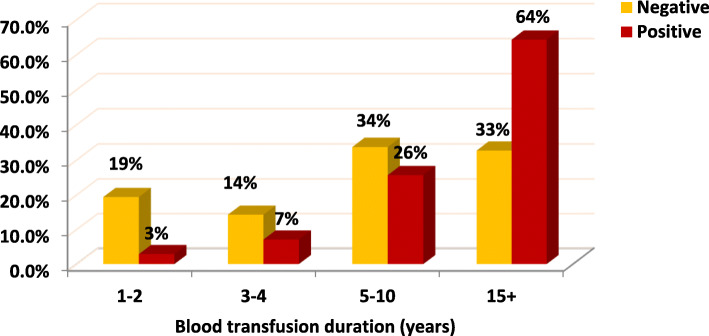
HCV Seropositive Rates among Different Transfusion Duration Groups

HCV seropositivity was significantly higher among older age patients, particularly > 15 years old as displayed in Fig. 2, those who received more blood transfusion (Fig. 3, Table 5) and also those with higher serum ferritin levels as shown in Table 5.

**Table 5:** Association of HCV Seropositivity with Demographic, Transfusion and Laboratory Variables

Variables	HCV Negative (***n*** = 407)	HCV Positive (***n*** = 70)	Test	***P*** value
**Gender No (%)**				
- Male	224 (55)	38 (54)	0.014^a^	0.927
- Female	183 (45)	32 (46)		
**Age (years)**				
- Mean (SD)	9.4 (±5.1)	13.1 (±5.2)	3.9^b^	0.001
**Age Categories (years) No (%)**				
- < 5	82 (20)	4 (5)		
- 5–9	146 (36)	14 (20)	29.6^a^	0.001
- 10–14	101 (25)	20 (29)		
- 15–18	78 (19)	32 (46)		
**Body mass index No (%)**				
- Underweight	250 (61)	30 (43)		
- Normal	144 (35)	38 (54)	17.3^a^	0.027
- Overweight	13 (4)	2 (3)		
**Transfusion Categories (years) No (%)**				
- 1–2	79 (19)	2 (3)		
- 3–4	58 (14)	5 (7)	29.9^a^	0.001
- 5–10	137 (34)	18 (26)		
- > 11	133 (33)	45 (64)		
**Transfusion Duration (year)**				
- Mean (SD)	9 (±2.9)	14 (±2.1)	2.7^b^	0.001
**Transfusion Frequency/years**				
- Mean (SD)	14.8 (±8.5)	19.5 (±9.7)	3.9^b^	0.001
**Serum Ferritin (ng/ml)**				
- Median	2800	3875	3.8^c^	0.001
- Range	488–4170	1300–9661		

^a^*Chi-square test,* ^b^ *Independent sample t-Test,* ^c^ *Mann-Whitney test*

Association between CMV seropositivity and different demographic and laboratory variables were evaluated as shown in Tables 6 & 7 where statistically significant higher data were reported for patients with higher leucocytic counts, higher alanine aminotransferase and higher serum ferritin values.

**Table 6:** Association between CMV Seropositivity with Demographic and Transfusion Variables

Variables	CMV Negative (***n*** = 445)	CMV Positive (***n*** = 32)	Test	***P*** value
**Gender No (%)**				
- Male	233 (52)	21 (66)	0.45^a^	0.49
- Female	212 (48)	11 (34)		
**Age (years)**				
- Mean (SD)	11.4 (±5.6)	11.2 (±3.9)	0.09^b^	0.92
**Age Categories (years) No (%)**				
- < 5	58 (13)	0 (0)		
- 5–9	116 (26)	16 (50)	2.1^a^	0.54
- 10–14	116 (26)	5 (17)		
- 15–18	155 (35)	11 (34)		
**Body mass index No (%)**				
- Underweight	211 (48)	16 (50)		
- Normal	233 (50)	16 (50)	0.15^a^	0.98
- Overweight	11 (2)	0 (0)		
**Hepatomegaly No (%)**	286 (64)	16 (50)	0.49^a^	0.48
**Spleen Status No (%)**				
- Splenomegaly	242 (53)	16 (50)	0.03^a^	0.86
- Splenectomized	140 (47)	16 (50)		
**Transfusion Categories (years) No (%)**				
- 1–2	43 (10)	0 (0)		
- 3–4	43 (10)	0 (0)	1.8^a^	0.62
- 5–10	136 (30)	16 (50)		
- > 11	223 (50)	16 (50)		
**Transfusion Duration (year)**				
- Mean (SD)	9.9 (±3.6)	8.5 (±3.1)	1.2^b^	0.56

^a^Chi-square test, ^b^ Independent sample t-Test

**Table 7:** Association between CMV Seropositivity and Laboratory Variables

Laboratory Investigation	CMV Negative (***n =*** 445)	CMV Positive (***n =*** 32)	Test	***P*** value
**Hb (gm/dL)**				
- Mean (SD)	7.9 (±1.0)	7.7 (±1.0)	0.46^b^	0.64
- Range	5.6–10	4.7–9.6		
**WBC’s**				
- Median	9.8	12	2.1^c^	0.03
- Range	4.6–8.7	2.4–24.9		
**Platelets (10**^**3**^**/dL)**				
- Median	301.7	397	0.10^c^	0.91
- Range	162–950	120–430		
**Serum Ferritin (ng/mL)**				
- Mean (SD)	2892 (±1749)	3515 (±2023)	1.9^b^	0.04
- Range	488–8724	601–9661		
**AST (IU/L)**				
- Median	59	85	11.8^c^	0.07
- Range	11–150	16–442		
**ALT (IU/L)**				
- Median	66	89	2^c^	0.04
- Range	6–180	13–292		
**Total Bilirubin (mg/dL)**				
- Mean (SD)	1.6 (±1)	1.7 (±1.3)	0.45^b^	0.65
- Range	1.2–3.9	1.3–4.7		

^b^Independent sample t-Test, ^c^ Mann-Whitney test, *SD* standard deviation

## Discussion

In the current study, we investigated the seroprevalence of the most common transfusion induced viral hepatitis; namely HCV, HBV and CMV; in a large cohort (477 patients) of multi-transfused children with inherited hemolytic anemia.

Β-thalassemia patients constituted 77% of our cohort. In Egypt, β-thalassemia is the most common chronic hemolytic anemia (85%) with more than one thousand expected affected cases born yearly [13]. Angelucci et al. 2016, reported β-thalassemia as an important public-health challenge in their Italian cohort. In the 114 treatment centers surveyed, a total of 7080 β-thalassemia patients were reported [14].

Globally, until early 1990s HCV used to infect a great proportion of transfusion–dependent hereditary hemolytic anemia patients. However, establishment of blood donner’s screening programs has dramatically reduced this infection rate [15].

Egypt was one of the highest prevalence countries for HCV. Treatment of HCV in Egypt has become one of the top national priorities since 2007. Mass HCV treatment program had started using Pegylated interferon and ribavirin between 2007 and 2014. Yet, with the development of highly-effective direct acting antivirals (DAAs) for HCV, The Egyptian National Committee for the Control of Viral Hepatitis launched campaign to provide Egyptian HCV patients with DAAs at no cost aiming at eliminating HCV infection completely by 2023 [16]. Significant reduction in Egyptian HCV prevalence was recorded after mass treatment campaigns, In 1996, the HCV seroprevalence was > 40% among adults, whereas in 2008, the Demographic Health Survey (DHS) showed a seroprevalence of 14.7%, and The latest DHS in 2015 reported a seroprevalence of 10% and viremic prevalence of 7% [17].

The current research has its unique peculiarity compared to the previously published Egyptian studies. As it is carried out after the implementation of HCV screening and DAAs treatment strategies, it probably reflects the direct influences of such modalities on HCV seroprevalence rate. The PCR confirmed HCV seroprevalence in our cohort of multi-transfused hemolytic anemia patients was 14.7%; compared to seroprevalence rate as high as 58% in remote Egyptian study [18].

Previously reported HCV seroprevalence rates were much higher; ranging from 60.6% [19], 51.7% [20], 48% [21], 40% [9] to 37.11% [7] for Egyptian thalassemia children in different studies. The lower reported rate in our study is largely attributed to the aforementioned preventive strategies. This is confirmed by the findings that, around 20% of our patients (86 patients), were younger than 5 years old, and were born after program implementation, they displayed a significantly lower HCV seropositivity rate (5.7%) as compared with older age groups in our cohort. This will definitely improves their future long term follow up particularly the hepatic complications. Bonifazi and coworkers in their Italian, largely adult study, found that HCV was responsible for 88.5% of hepatic complication in their cohort [22].

In 2010, Alavian published a systematic review on HCV seroprevalence among thalassemia patients in Eastern Mediterranean Countries with much higher rates than that detected in our study; 18, 45, 63 and 69% in Iran, Pakistan, Saudi Arabia and Egypt, respectively [23]. Once again, their study was 10 years back, before HCV mass screening and treatment campaign implementation in Egypt.

In India; higher seroprevalence rates were reported by Bhavsar and his team [24] and Jian and coworkers [25] in 2011 and 2012 respectively, with 18 and 25% HCV Ab seroprevalence and 6 and 1.04% HBsAg positivity. These findings could be partly explained by their largely adult cohort.

Mirmomen et al. in his 732 Iranian patients with thalassemia agreed with our results but with higher HCV and HBV seropositivity; 19.3 and 1.5% respectively; which can be explained by the earlier timing of their study in 2006 before the recent advances in blood donation screening and hepatitis viruses diagnosis and recent oral antiviral treatment [26].

In agreement with other authors, the main associated factors with HCV seropositivity in our study were older age [7, 9, 20], higher number of transfusions [7, 9], longer disease and transfusion duration [7].

In 2011, Omar and his colleagues found that higher serum ferritin was the most significant risk factors in the HCV positive (51.7%) patients of his 174 cohort of multi-transfused thalassemic patients from Cairo, Egypt [20]. Similarly, Mahmoud and his colleagues reported similar results in his study on 97 thalassemia children from Sohag and Minia Universities; Egypt; with higher serum ferritin, and liver enzymes being the most correlated factors with seropositivity [7]. Our results also declared serum ferritin as a significant associated factor to HCV seropositivity. The chief source of iron overload in thalassemia major is transfused blood [27]. S. Ferritin may represent an indirect indicator of the total amount of transfused blood and the subsequent increased risk of capturing HCV infection.

Different types of iron chelation therapy were used in all our patients but unfortunately 65% of them were noncompliant. Del Vecchio & coresearchers in their study on Deferiprone in their Italian thalassemia major patients, had observed that iron chelation therapy caused a recovery of immune status and cytokine pattern which had both been slightly activated before treatment [28].

Regarding CMV seropositivity in our cohort, 32 patients (6.7%) were positive despite negative pretransfusion results. Higher leucocytic count, mean serum ferritin, and ALT were the significantly associated factors with seropositivity. Lower rates of 4.12% were reported from other Egyptian studies with higher serum ferritin and ALT being the most correlated factors [7].

The universal CMV-IgM screening and leucoreduction carried in many western countries, markedly eliminated risk of CMV transmission from donors’ blood [29]. This technique still not universally applied in Egypt which might explain the risk for transfusion transmitted CMV. Twenty-two out of the 32 CMV positive patients, were positive to HCV as well, a finding that points to a common route of acquisition of both, mostly post-transfusion.

Egypt has implemented compulsory routine infant hepatitis B virus vaccination in 1992. As 90% of infants infected at birth will develop chronic hepatitis and associated morbidities, in 2017 health authorities in Egypt added HBV birth dose to be administered to all neonates during the first 48 h of life regardless their maternal HbsAg status. This regimen aimed at prevention of all routes of HBV infection; peri-natally and post-natalley, and ensuring long term protection against HBV infection [30, 31]. Egyptian study by Salama and co-workers documented the high long- term protective efficacy of HBV vaccine, even after waning of anti-Hbs antibody with time, due to the presence of potent immune memory that respond by powerful anamnestic reaction on exposure to HBV [32]. No single case of HBV infection could be recorded among multi-transfused children in the current study, either at diagnosis or after 1–17 years of the illness and multiple transfusion. This finding appreciate the high prolonged protective efficacy of the current HBV vaccination program and highlight the paramount importance of its administration to all multi-transfused patients if previously missed.

Similarly, in 2011, Omar and co-workers; Cairo-Egypt; had none of their hemophilia patients to be HBsAg +ve [20]. Contradictory data were reported by authors from Mansoura [9], Qena [19], Sohag and Minia [7]; Egypt; with 29, 12, and 4.12% HBsAg positivity in their multi-blood-transfused cohorts which may be explained by the older age in their cohorts being mostly adults born before the implementation of compulsory HBV vaccination for infants in Egypt.

In countries where effective blood screening programs have been implemented, the risk of transmission of blood born hepatitis viruses has been reduced dramatically over the last 20 years. Despite promising figure displayed in our cohort, HCV transmission will presents a significant problem to multi-transfused patients as long as “HCV-ab” is the used screening test for blood donors. The detection of HCV RNA should be used instead to reduce the risk of HCV transmission through the transfusion of infected blood donated during the window period of antigen and antibody assays [33]*.*

## Conclusions

None of studied cohort was positive for HBV. Compared to previous studies released from Egypt, transfusion transmitted HCV in multi-transfused hemolytic anemia pediatric patients has significantly reduced due to the national mass screening and treatment strategies implemented by Egyptian health authorities. CMV transfusion transmission risk is still a problem and needs call for immediate action. National implementation of universal CMV-IgM screening, leukoreduction and PCR screening for HCV in donors’ blood is mandatory step towards reduction of transfusion transmitted viruses in multi-transfused patients.

## Data Availability

All data and materials related to the study are included in the current manuscript.

## Abbreviations

HCV: Hepatitis-C-Virus
HBV: Hepatitis-B-Virus
CMV: Cytomegalovirus
HBsAg: Hepatitis B surface antigen
HCVab: Hepatitis C virus antibodies
IgM: Immunoglobulin M
PCR: Polymerase chain reaction
RBC: Red blood cell
BMI: Body mass index
CBC: Complete blood count
ELISA: Enzyme-linked immunosorbent assay
SD: Standard Deviation
ALT: Alanine aminotransferase
AST: Aspartate aminotransferase
Hb: Hemoglobin
WBCs: White blood cells
